# Supplementary material for: Comparison of IncK-blaCMY-2 Plasmids in Extended-Spectrum Cephalosporin-Resistant Escherichia coli Isolated from Poultry and Humans in Denmark, Finland, and Germany
Source: Antibiotics (Basel). 2024 Apr 10;13(4):349. doi: 10.3390/antibiotics13040349 (PMC11047599; doi:10.3390/antibiotics13040349)
Supplement: Supplementary file 1 [file antibiotics-13-00349-s001.zip › Fig S1. Comparison of Danish plasmids.pdf]

(a)

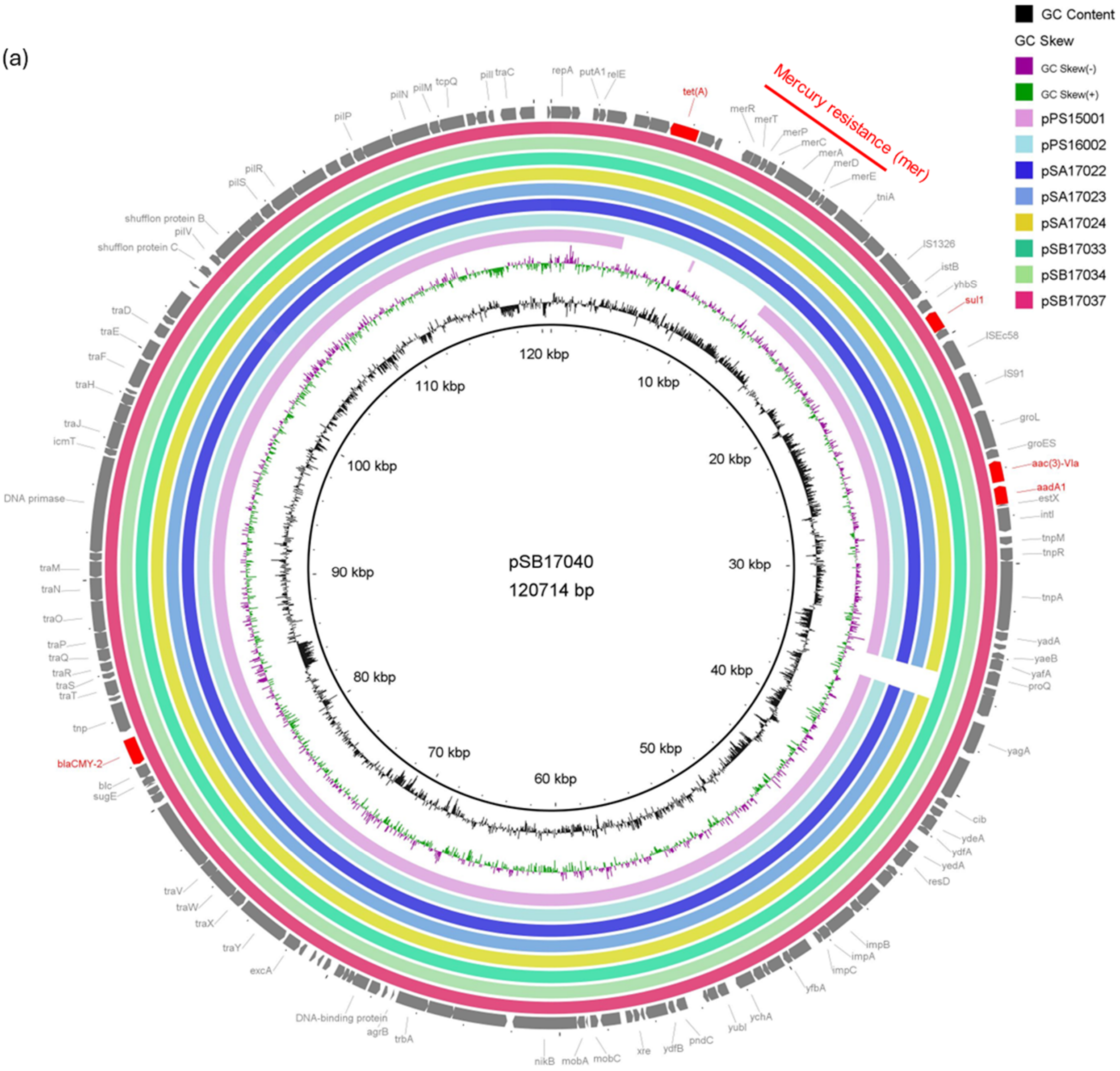

GC Content
GC Skew
GC Skew(-)
GC Skew(+)
pPS15001
pPS16002
pSA17022
pSA17023
pSA17024
pSB17033
pSB17034
pSB17037
Mercury resistance (mer)
pSB17040
120714 bp
120 kbp
10 kbp
20 kbp
30 kbp
40 kbp
50 kbp
60 kbp
70 kbp
80 kbp
90 kbp
100 kbp
110 kbp
tet(A)
sul1
aac(3)-VIa
aadA1
blaCMY-2

(b)

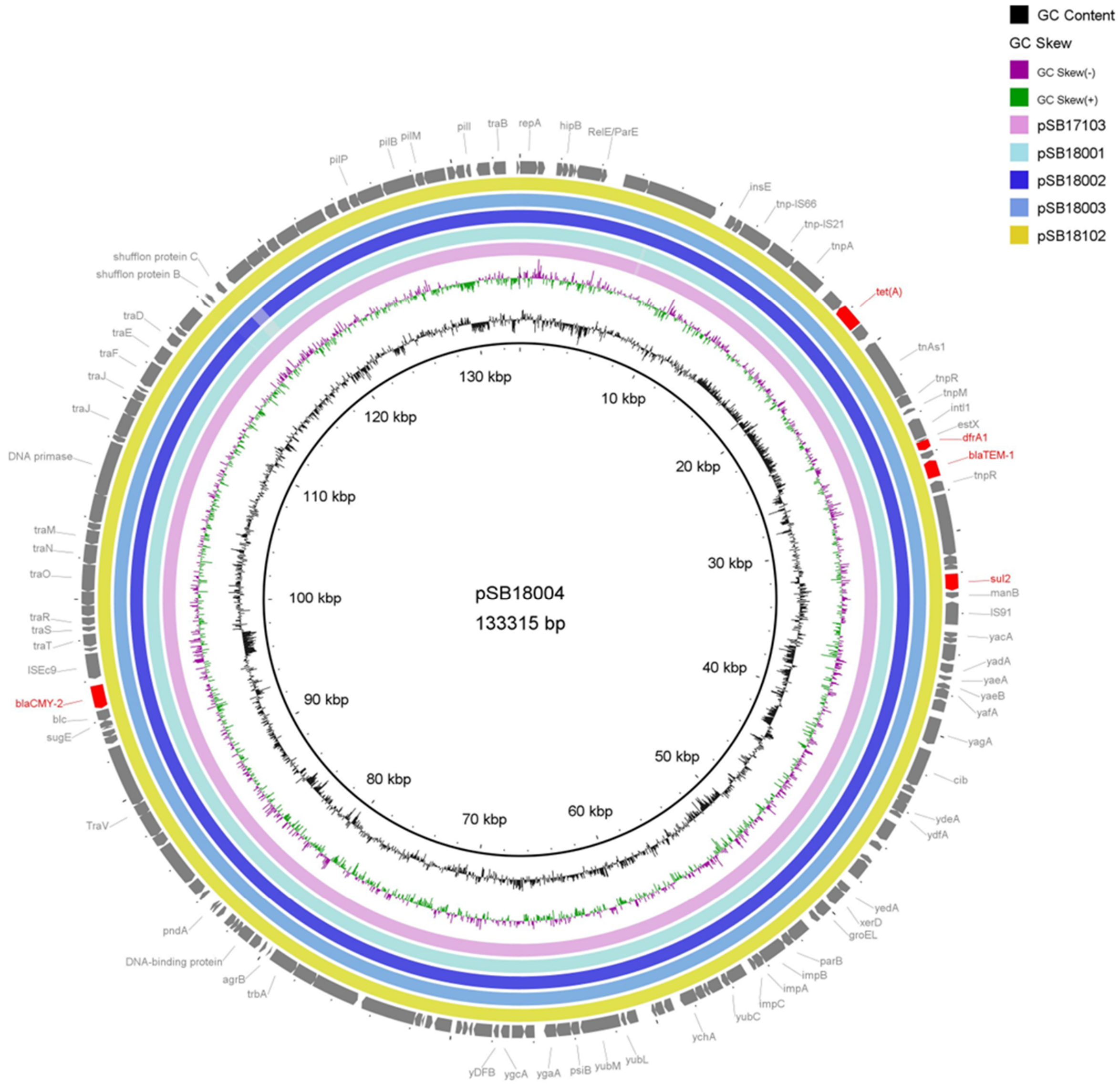
GC Content
GC Skew
GC Skew(-)
GC Skew(+)
pSB17103
pSB18001
pSB18002
pSB18003
pSB18102
pSB18004
133315 bp
10 kbp
20 kbp
30 kbp
40 kbp
50 kbp
60 kbp
70 kbp
80 kbp
90 kbp
100 kbp
110 kbp
120 kbp
130 kbp
repA
hipB
RelE/ParE
insE
tnp-IS66
tnp-IS21
tnpA
tet(A)
tnAs1
tnpR
tnpM
intI1
estX
dfrA1
blaTEM-1
tnpR
sul2
manB
IS91
yacA
yadA
yaeA
yaeB
yafA
yagA
cib
ydeA
ydfA
yedA
xerD
groEL
parB
impB
impA
impC
yubC
ychA
yubL
yubM
psiB
ygaA
ygcA
yDFB
trbA
agrB
DNA-binding protein
pndA
TraV
sugE
blc
blaCMY-2
ISEc9
traT
traS
traR
traO
traN
traM
DNA primase
traJ
traJ
traF
traE
traD
shufflon protein B
shufflon protein C
pilP
pilB
pilM
pilI
traB

(c)

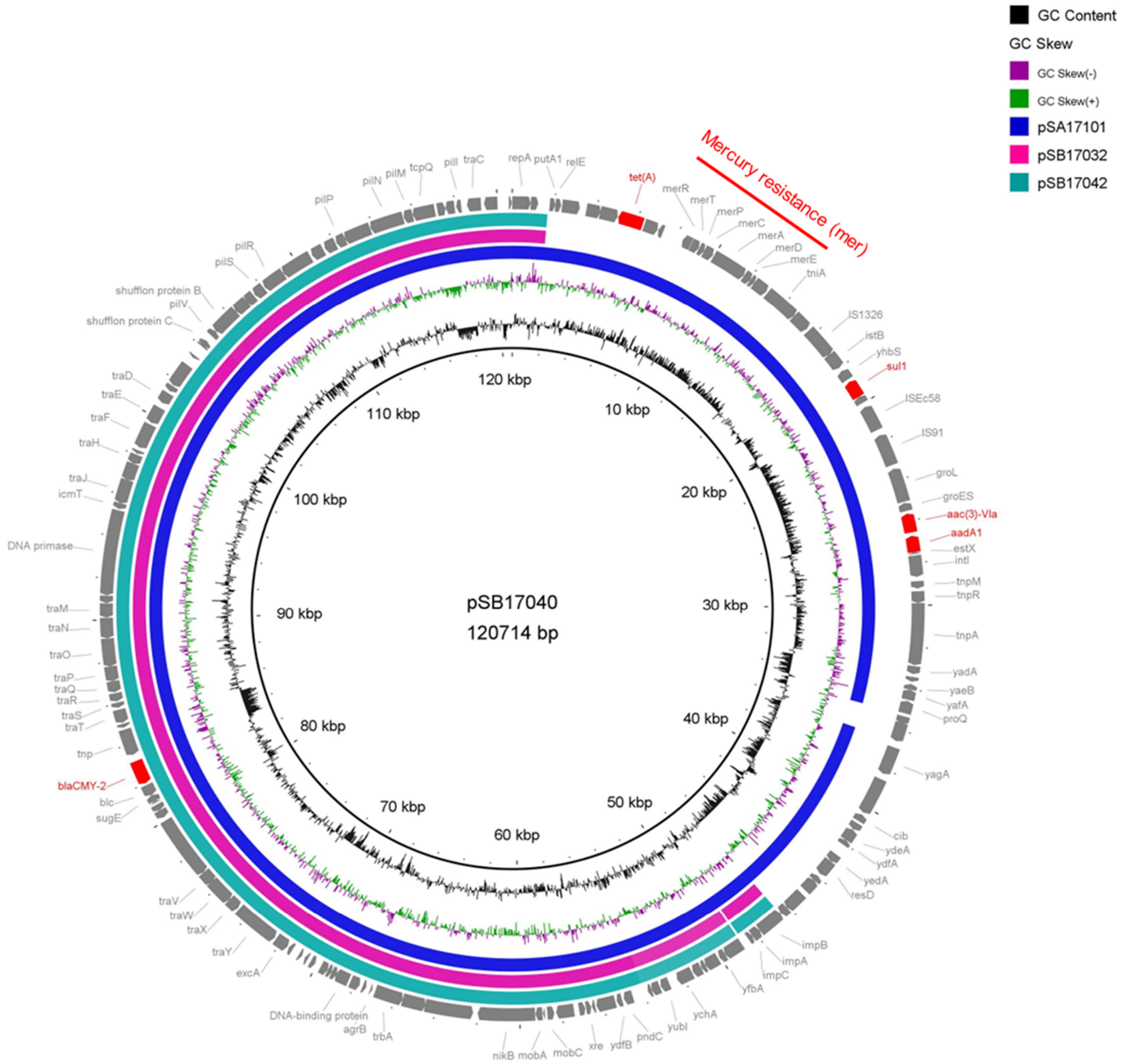
GC Content
GC Skew
GC Skew(-)
GC Skew(+)
pSA17101
pSB17032
pSB17042
Mercury resistance (mer)
pSB17040
120714 bp
120 kbp
10 kbp
20 kbp
30 kbp
40 kbp
50 kbp
60 kbp
70 kbp
80 kbp
90 kbp
100 kbp
110 kbp
repA
putA1
relE
tet(A)
merR
merT
merP
merC
merA
merD
merE
tniA
IS1326
istB
yhbS
sul1
ISEc58
IS91
groL
groES
aac(3)-VIa
aadA1
estX
intI
tnpM
tnpR
tnpA
yadA
yaeB
yafA
proQ
yagA
cib
ydeA
ydfA
yedA
resD
impB
impA
impC
yfbA
ychA
yubI
pndC
ydfB
xre
mobC
nikB mobA
trbA
agrB
DNA-binding protein
excA
traY
traX
traW
traV
sugE
blc
blaCMY-2
tnp
traT
traS
traR
traQ
traP
traO
traN
traM
DNA primase
icmT
traJ
traH
traF
traE
traD
shufflon protein C
pilV
shufflon protein B
pilS
pilR
pilP
pilN
pilM
tcpQ
pilI
traC

(d)

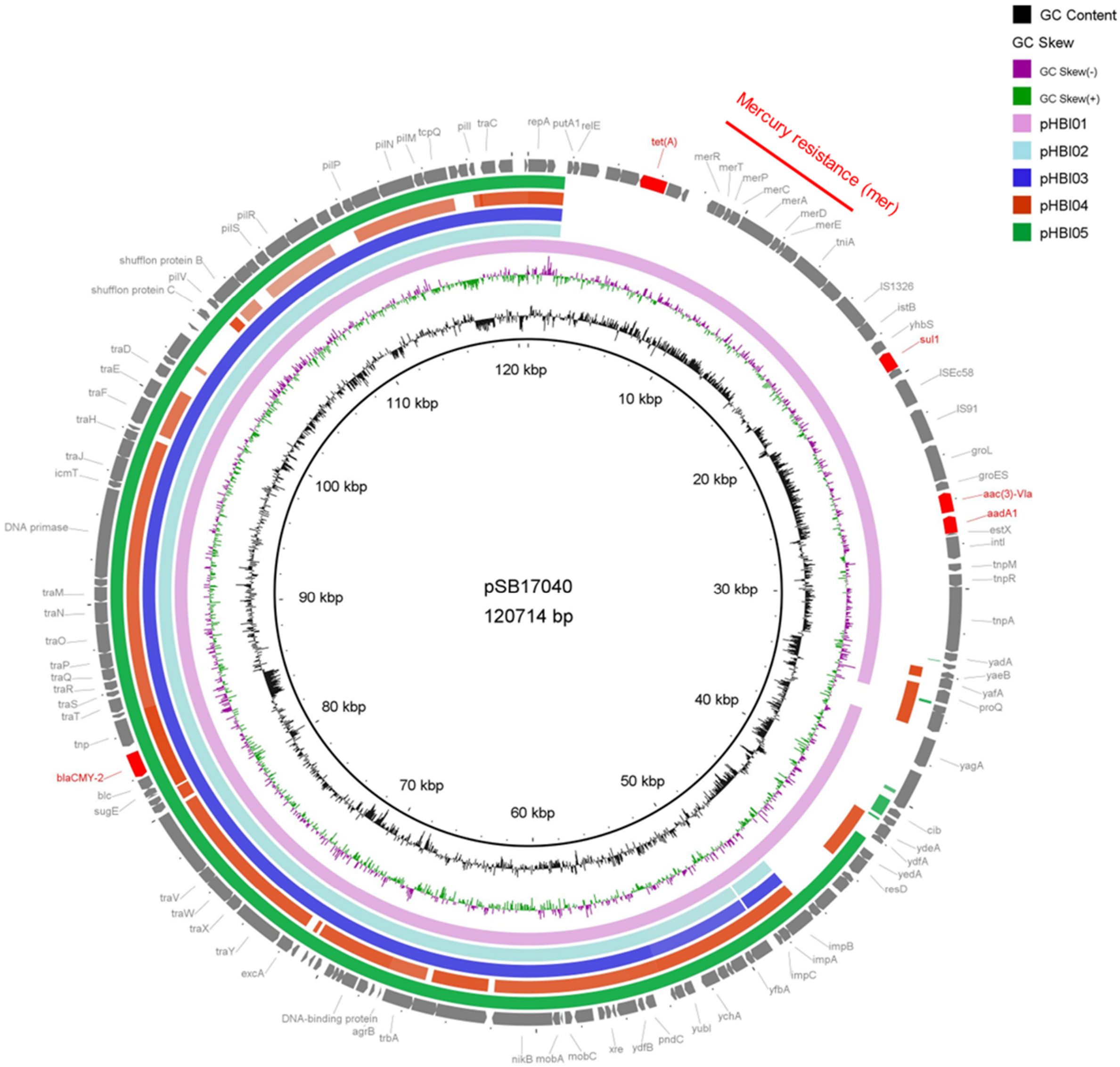

Supplementary Figure S1. Circular comparison analysis of Danish IncK-*bla*$_{CMY-2}$ plasmids. GC content and GC skew are shown in the inner map. The predicted coding sequence of the plasmid within the circle is annotated in the outer ring with resistance genes highlighted in red. (a) Comparison of nine IncK-*bla*$_{CMY-2}$ plasmids in ST429 ESC-resistant *E. coli* from the Danish poultry system and slaughterhouses using pSB17040 as the reference. (b) Comparison of six IncK-*bla*$_{CMY-2}$ plasmids present in ST1286 ESC-resistant *E. coli* from Danish slaughterhouses using pSB18004 as the reference. (c) Comparison of three IncK-*bla*$_{CMY-2}$ plasmids in ST162, ST350 and ST57 ESC-resistant *E. coli* from Danish slaughterhouses using pSB17040 as the referene. (d) Comparison of five IncK-*bla*$_{CMY-2}$ plasmids in different STs (ST429, ST69, ST131, ST95, ST12) ESC-resistant *E. coli* from Danish human clinical samples with the poultry associated pSB17040 reference plasmid.
