# Supplementary material for: Comparison of IncK-blaCMY-2 Plasmids in Extended-Spectrum Cephalosporin-Resistant Escherichia coli Isolated from Poultry and Humans in Denmark, Finland, and Germany
Source: Antibiotics (Basel). 2024 Apr 10;13(4):349. doi: 10.3390/antibiotics13040349 (PMC11047599; doi:10.3390/antibiotics13040349)
Supplement: Supplementary file 1 [file antibiotics-13-00349-s001.zip › Fig S2. Comparison of Danish and international plasmids.pdf]

(a)

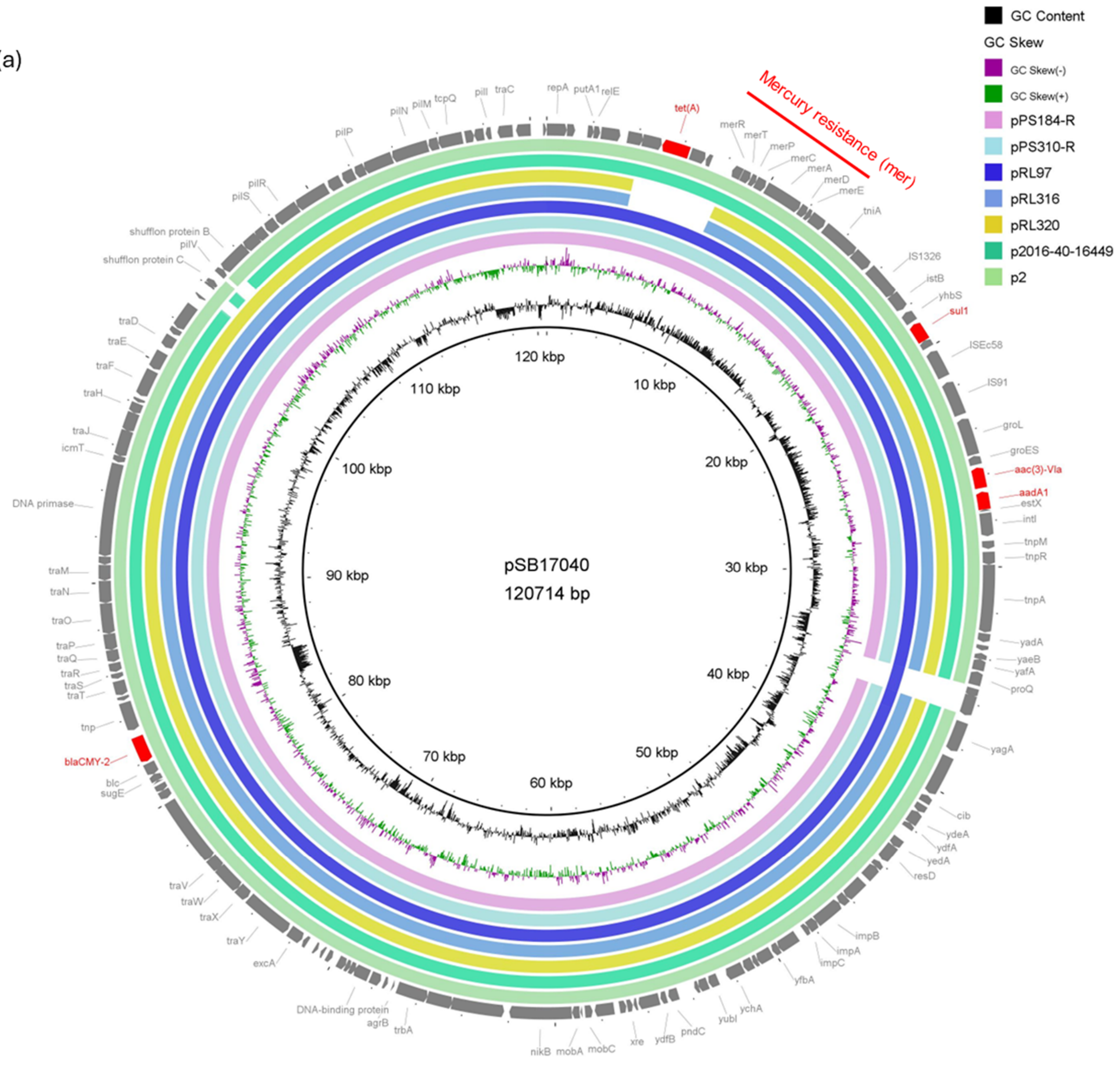
GC Content
GC Skew
GC Skew(-)
GC Skew(+)
pPS184-R
pPS310-R
pRL97
pRL316
pRL320
p2016-40-16449
p2
Mercury resistance (mer)
pSB17040
120714 bp
120 kbp
10 kbp
20 kbp
30 kbp
40 kbp
50 kbp
60 kbp
70 kbp
80 kbp
90 kbp
100 kbp
110 kbp
repA
putA1
relE
tet(A)
merR
merT
merP
merC
merA
merD
merE
tniA
IS1326
istB
yhbS
sul1
ISEc58
IS91
groL
groES
aac(3)-VIa
aadA1
estX
intI
tnpM
tnpR
tnpA
yadA
yaeB
yafA
proQ
yagA
cib
ydeA
ydfA
yedA
resD
impB
impA
impC
yfbA
ychA
yubI
pndC
ydfB
xre
mobC
mobA
nikB
trbA
agrB
DNA-binding protein
excA
traY
traX
traW
traV
sugE
blc
blaCMY-2
tnp
traT
traS
traR
traQ
traP
traO
traN
traM
DNA primase
icmT
traJ
traH
traF
traE
traD
shufflon protein C
pilV
shufflon protein B
pilS
pilR
pilP
pilN
pilM
tcpQ
pilI
traC

(b)

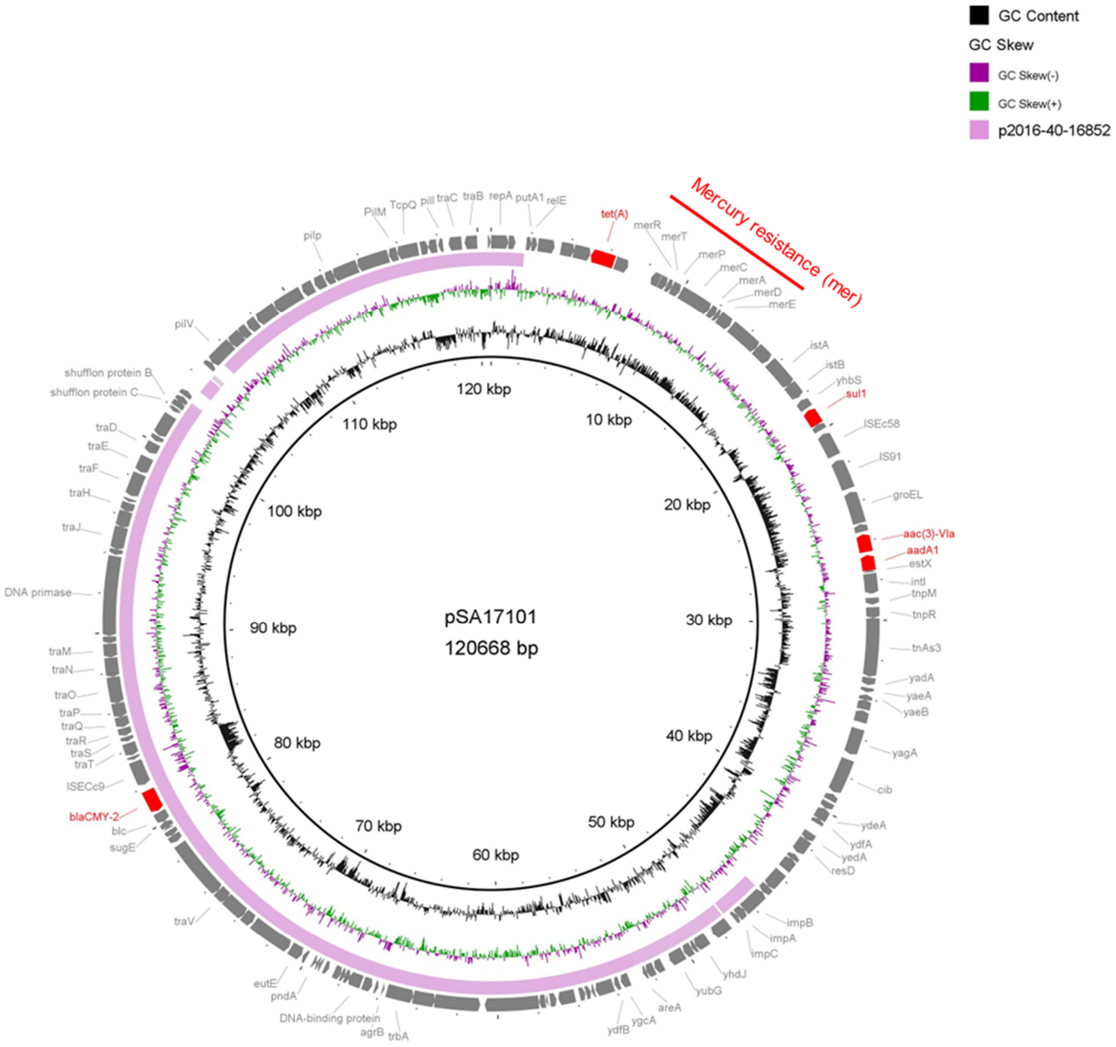

Supplementary Figure S2. Circular comparison analysis of Danish IncK-*bla*$_{CMY-2}$ plasmids with plasmids from other countries. GC content and GC skew are shown in the inner map. The predicted coding sequence of the plasmid within the circle is annotated in the outer ring with resistance genes highlighted in red. (a) Seven IncK-*bla*$_{CMY-2}$ plasmids in ST429 ESC-resistant *E. coli* from other countries were compared with the Danish pSB17040. Among them were ones characterized in the present study; pPS184-R and pPS310-R from Finish poultry, and pRL-97, pRL310 and pRL320 from German chicken meat. Previously published poultry associated plasmids from Norway (p2016-40-16449, Mo et al., 2021), and USA (p2, Feng et al., 2023) were also included. (b) Comparison of the Danish pSA17101 with an IncK-*bla*$_{CMY-2}$ plasmid present in a ST162 ESC-resistant *E. coli* from Norwegian poultry.
