# Supplementary material for: Comparison of IncK-blaCMY-2 Plasmids in Extended-Spectrum Cephalosporin-Resistant Escherichia coli Isolated from Poultry and Humans in Denmark, Finland, and Germany
Source: Antibiotics (Basel). 2024 Apr 10;13(4):349. doi: 10.3390/antibiotics13040349 (PMC11047599; doi:10.3390/antibiotics13040349)
Supplement: Supplementary file 1 [file antibiotics-13-00349-s001.zip › FigS3a-c. Minimum spanning trees of the E. coli ST429.pdf]

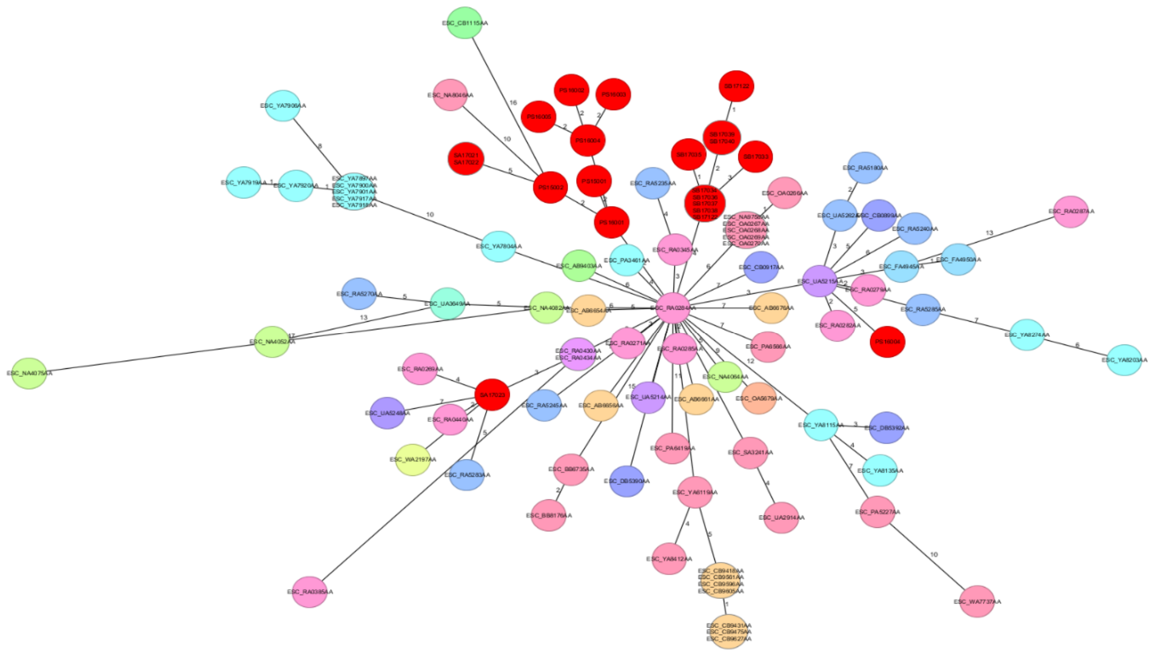

(**a**)

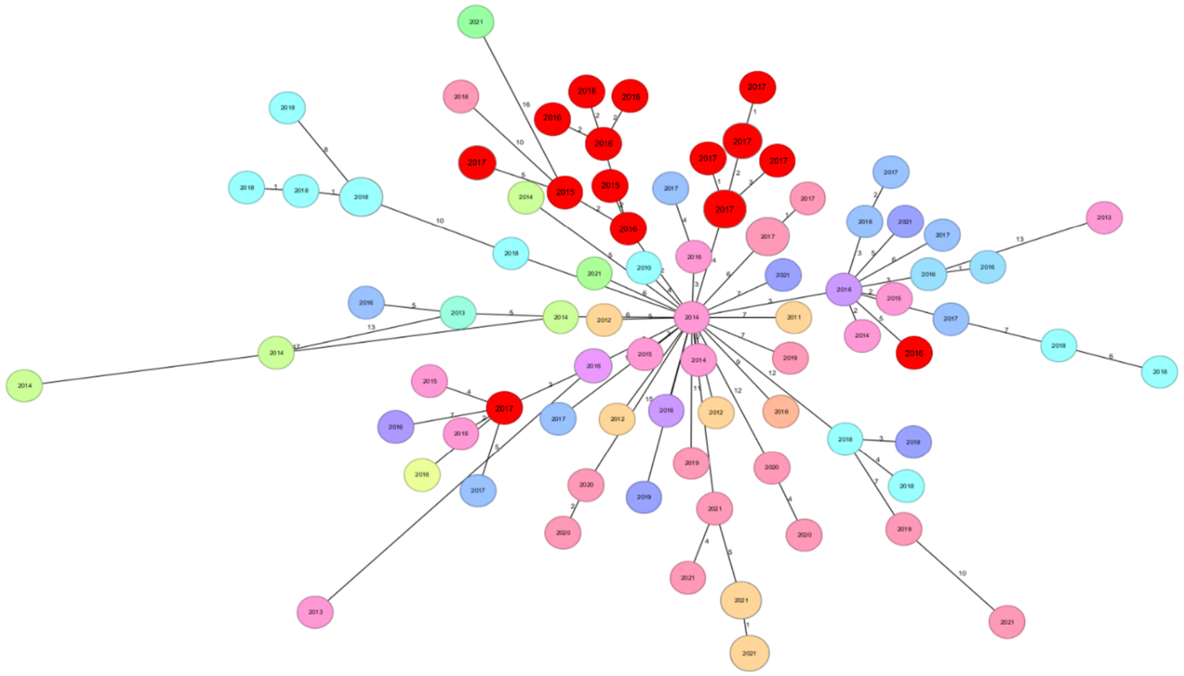

(**b**)

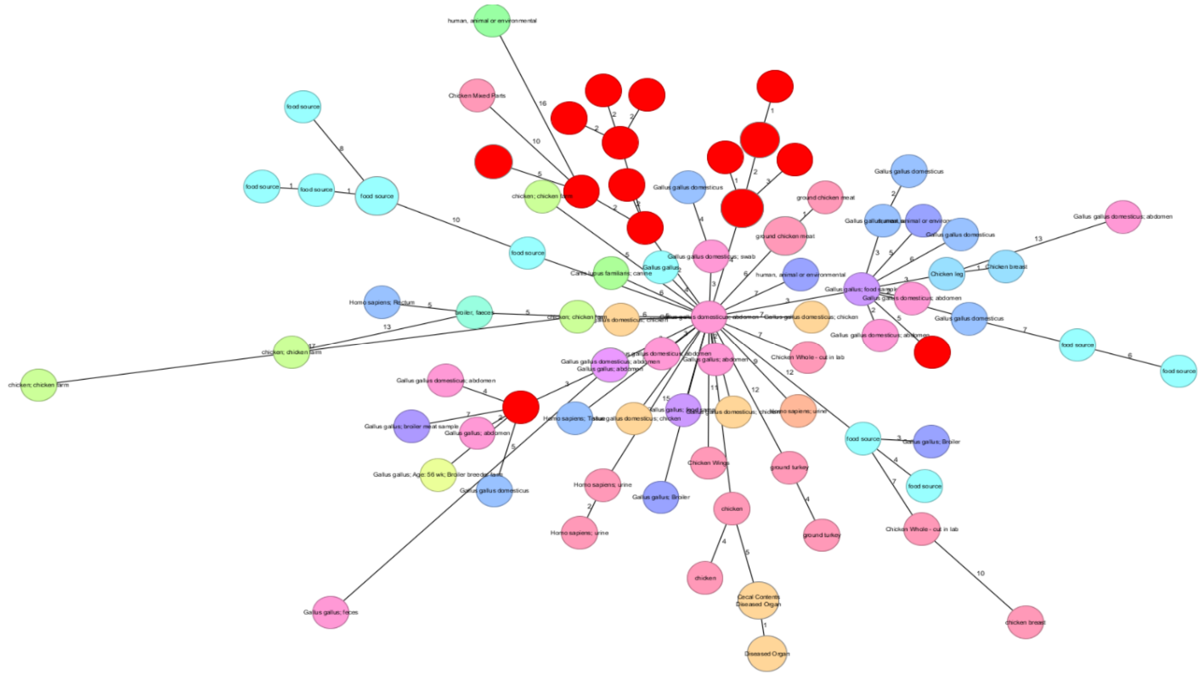

(**c**)

Figure S3. (**a**) Minimum spanning trees of the *E. coli* ST429 with information regarding isolate numbers. (**b**) Minimum spanning trees of the *E. coli* ST429 with information regarding year of isolation. (**c**) Minimum spanning trees of the *E. coli* ST429 with information regarding source. All Danish isolated (red circles) are from chicken production.
